# Replacing vaccine paper package inserts: a multi-country questionnaire study on the acceptability of an electronic replacement in different target groups

**DOI:** 10.1186/s12889-022-12510-8

**Published:** 2022-01-24

**Authors:** Martina Bamberger, Hans De Loof, Charlotte Marstboom, Stéphanie Oury, Paolo Bonanni, Odile Launay, Mira Kojouharova, Pierre Van Damme

**Affiliations:** 1 Centre for the Evaluation of Vaccination, Vaccine and Infectious Disease Institute, University of Antwerp, Universiteitsplein 1, 2610 Antwerp, Belgium; 2 Laboratory of Physiopharmacology, University of Antwerp, Universiteitsplein 1, 2610 Antwerp, Belgium; 3 Department of Health Sciences, University of Florence, Florence, Italy; 4 CIC Cochin Pasteur, Paris, France; 5 Epidemiology consultant, Sofia, Bulgaria

**Keywords:** Vaccine, Leaflet, Patient health questionnaire, Culturally appropriate technology, Pregnancy, Parents

## Abstract

**Background:**

In the European Union it is mandatory to include paper package leaflets (PPL) with all medicines, including vaccines, to inform the recipient. However, it is difficult to meet the necessity for localized PPLs in each of the 24 official European languages. Replacing PPLs with electronic versions offers many advantages including redistribution across nations, reduced storage space, accessibility by the visually impaired, easily updated information or the addition of video content. We wanted to assess the attitudes of patients (vaccine recipients or their parents) to the potential of replacing PPL with electronic versions.

**Methods:**

We surveyed vaccinees or their parents in four European countries—Belgium, Italy, Bulgaria and France—for their actual use of vaccine PPLs and their opinions about switching to an electronic package leaflet. Our survey was conducted online because of the COVID-19 pandemic and resulted in 2518 responses to a questionnaire targeted at three specific groups with particular information needs: parents of young children, pregnant women and the elderly (≥ 60 years).

**Results:**

Our main findings are that currently vaccine PPLs are rarely used and frequently unavailable for the vaccinee. Across the four countries surveyed 55–82% of vaccinees would accept an electronic version, as did 64% when there was an option to request a printout of the leaflet.

**Conclusions:**

We found that switching to electronic versions of vaccine PPLs is an acceptable alternative for the public, potentially increasing the quality and amount of information reaching vaccinees while eliminating some barriers to redistribution of vaccines between countries.

**Supplementary Information:**

The online version contains supplementary material available at 10.1186/s12889-022-12510-8.

## Background

There is little doubt among most of the general public and certainly among scientists of the benefits of vaccines [1]. Nonetheless, the phenomenon of ‘vaccine hesitancy’ [2] is just as real and is recognized by the World Health Organization as a major public health threat [3]. Vaccinees or their parents are exposed to all kinds of information from a variety of sources, not all of which are regulated [4–8]. Therefore, a three-pronged strategy in a global plan of action included a proposal to transform this informational environment to allow for the provision of scientifically valid information in an evidence-based manner [9], under the responsibility of numerous actors [6].

Governments, healthcare providers, regulatory bodies and responsible media organizations need to reappraise their means of communication to counter the anti-vaccine lobby. There is evidence that the need for information by vaccinees or parents of vaccinees is only partially met [10], with uncertainty on how to most effectively counteract vaccine hesitancy which causes low vaccination rates [11]. In the European Union, the established means of communicating such information about medicines to recipients is through the paper package leaflet (PPL) or package insert [12–14]. In the United States the PPL is intended to inform the healthcare professional dispensing the medication, but in Europe the PPL is intended to inform the lay public, i.e. the recipient or parents/guardians of children, as well as the user on the safe use of the medication. Inclusion of a PPL based on the Summary of Product Characteristic in the medicine packaging is a legal requirement for all medicines, including vaccines [15–17]. Although the PPL should not be the sole way to inform the patient [18, 19], and despite some improvement and the existence of guidelines [13], several problems remain [20–25]. There is evidence that the current leaflets for vaccines can be improved [26, 27].

One potential improvement would be the adoption of strategies involving modern digital information technologies, the most frequently means exploited to communicate messages supporting vaccine hesitancy [27–31]. One suggestion is the development of electronic leaflets, scanning a code on the vaccine container with a smartphone or tablet to display the information typically provided in the PPL [32, 33]. This could be enhanced with video material [34], made available in different languages, or provided as spoken text for the visually impaired [35]. An electronic system is already operational in Spain for medicines [36]. This also offers the opportunity to incorporate the most up-to-date information very quickly [37].

In Europe medicine and vaccine PPLs must be provided in the official language(s) of the country where they are marketed [16], limiting redistribution among EU Member States to address local needs or to alleviate shortages quickly. Use of electronic package leaflets (ePLs) in all 24 EU languages would contribute to increase vaccine supply flexibility across the EU.

In this context we studied the access and use of current PPLs in a diverse set of vaccinees in different European countries and assessed opinions and attitudes to an electronic replacement. Our main aim was to better understand how vaccinees come into contact with PPL and what they think of the current paper-based approach, while also probing attitudes about partial or full substitution of paper-based information with an electronic leaflet.

## Methods

### Participants

We designed and implemented a survey about PPLs in broad, diverse samples of populations from four different European countries (Belgium, France, Italy and Bulgaria) as well as three specific “target groups”. The countries were selected to reflect the diversity in the structure of healthcare, web access and vaccine pricing policies in Europe, e.g. in France and Belgium many vaccinees bring along their vaccine to the healthcare provider [38]. Specified “target groups” were pregnant women, irrespective of gestational age, parents present during the vaccination of their child (< 12 years), and the elderly (≥ 60 years). Pregnant women and parents were assumed to have a greater desire for information about the safety or effectiveness of vaccines [39–41] while the elderly were assumed to have lower digital literacy [42–44]. Our questionnaire was about the most recent vaccination event, which must have taken place within the last two years. We aimed at 100 respondents in each target group in each country.

When initiating the project we had planned for in person contacts at hospitals, pharmacies, kindergartens and other public places to complete the questionnaires, but with the onset of the COVID-19 pandemic at the beginning of 2020 we adapted our survey to an online setting [45]. As data collection would have been done electronically this adjustment had only a limited impact on the structure and content of the survey and mainly affected the distribution of the survey as described for each country below.

**Fig. 1:**
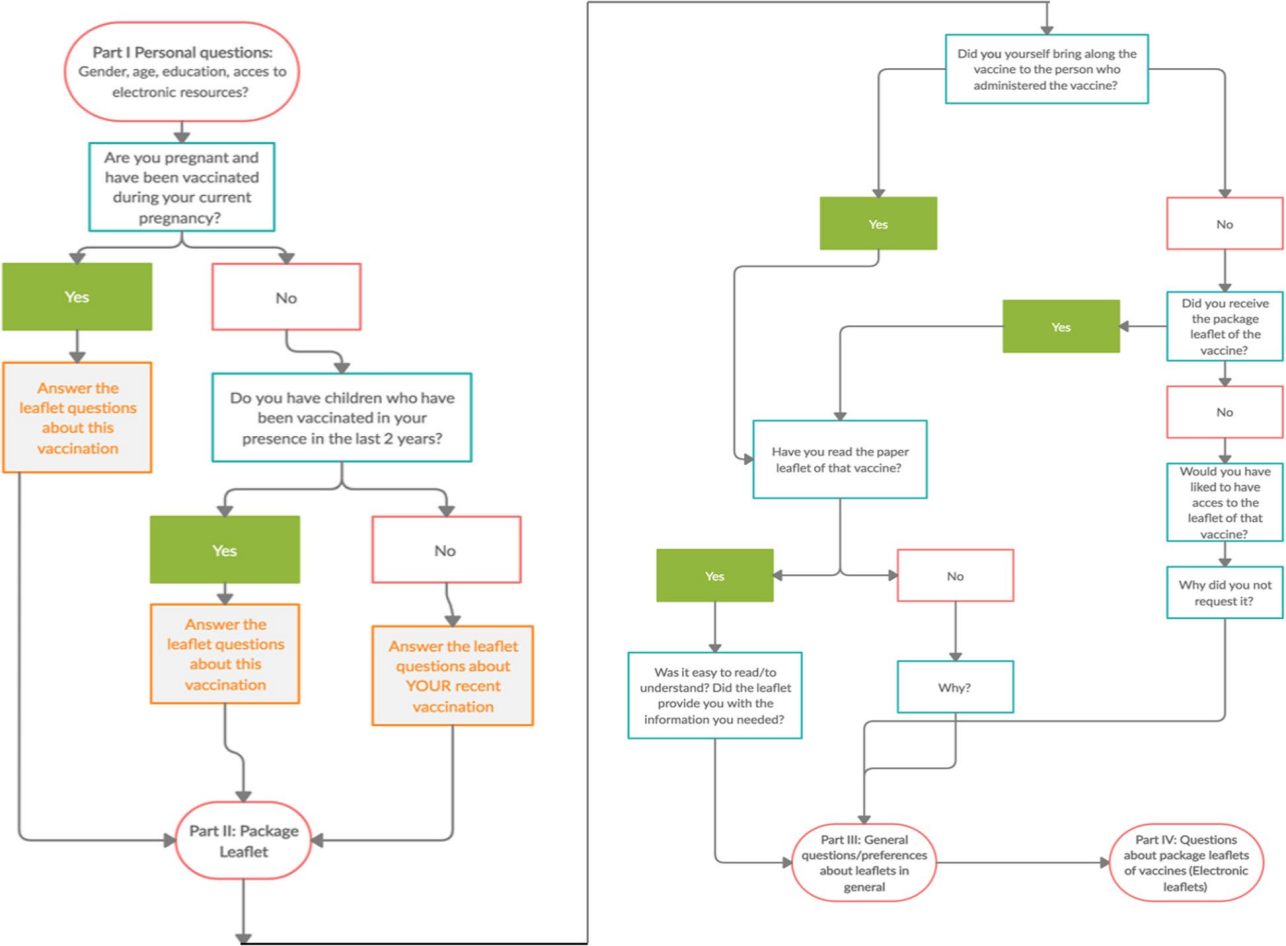
Questionnaire flow chart

The iterative design and development of the questionnaire was carried out on an English-language version before involving collaborators in the four countries to obtain a single questionnaire to be used in the different settings, including the necessary questions to separate the participants into the different target groups. The study setup and the English questionnaire (see Supplementary Material) were approved by the ethics committee of the University of Antwerp Approval number 20/07/079 UZA/University Antwerp Ethics committee after which translations into Dutch, Italian, Bulgarian and French were made, and approval was obtained from the Ethics Committee of the Area vasta Centro at Careggi University Hospital, Florence for Italy (CEAVC 16,714/2020), and the Comité d’éthique de la recherche AP-HP.5 (CERAPHP.5), Hôpitaux de Paris Centre, for France (#00011928). Ethical approval was not sought in Bulgaria as the Ethics Committee for Clinical Trials of the Bulgarian Ministry of Health only assesses studies with medicinal products. About ten subjects in each country, who were excluded from participating, validated final versions of the questionnaire for length and comprehensibility. The full questionnaire was in four parts, with 30 questions including YES/NO questions, open questions and multiple-choice questions. The time for completion was determined to be about 10 min. A flowchart of the survey is given in Fig. 1.

All data was collected in Qualtrics XM [46] and subsequently exported to IBM SPSS Statistics 26 [47] for further analysis. Unfinished questionnaires or any questionnaires in which the free form input was in conflict with the inclusion criteria were not analyzed. Participants were included in the pregnant or parent target groups if they answered positively to Q7 or Q8, respectively. People answering no to the above questions and who were 60 years of age or older (Q3) were included in the elderly group. Responses from 443 people who responded to the questionnaire but were not part of any target group, e.g. younger than 60 or not pregnant, following a recent personal vaccination, were not included in the analysis except for one additional analysis indicated in the Results section.

### Recruitment

While seeking data from about 100 individuals for each target group in each country recruitment inevitably had to be adapted to the local circumstances; in Bulgaria pregnant women or elderly people were not included as vaccination uptake is currently too low in these groups [48]. In Bulgaria a QR code linking to the questionnaire was distributed to parents with young children and, through snowball sampling, distributed further. It was also circulated in a number of closed online groups of parents with small children. In addition, health mediators working with parents from vulnerable communities in Bulgaria, helped to recruit additional survey respondents.

In France target groups were recruited by distributing the link among the personal contacts of the local academic investigator followed by snowball sampling. In addition, telephone interviews were conducted using the database of volunteers for the Investigational Clinical Centre (CIC) at the Cochin Hospital in Paris. To achieve 100 people in each target group, we included 18 women who recently gave birth and were still present in the maternity unit in the pregnant group.

In Italy pregnant women were recruited from the gynecology clinics at the University-Hospital of Careggi (Florence). Due to COVID-19 plans to distribute the questionnaire for the two other target groups had to be amended and dissemination of the online version was facilitated through the Vaccinars in Toscana website (https://www.vaccinarsintoscana.org), its related Facebook page and through two national organizations: “Happy Ageing” and “Active Citizenship”. In addition, a group of residents of the Medical Specialization School of Hygiene and Preventive Medicine (University of Florence) helped to distribute the questionnaire through personal contacts and further snowball distribution.

In Belgium a small number of face-to-face interviews were performed before switching to the online version. The link to the questionnaire was spread through personal contacts, individual gynecologists and midwives, and pharmacy students of the University of Antwerp. Several organizations helped to reach different target groups: *SeniorenNet.be* (a general website for seniors), *Kind&Gezin, (*Flemish agency for Child and Family public health and welfare), *Kraamvogel* (a maternity care organization), *and Gezinsbond* (Flemish nonprofit organization supporting families). Due to a slow uptake of responses from pregnant women, in the last weeks of the survey the questionnaire was distributed via Facebook through specific groups including ‘*Zwanger en bevallen in Antwerpen’*, ‘*Zwanger/Mama tijdens corona’* in addition to some local community groups such as ‘*Ge zijt van Antwerpen*’ and ‘*Ge zijt van Essen’*.

Data from each country and each target group were analyzed separately. There are sections of the questionnaire (part II, questions 10 through 19) where branching occurred and, depending on the answer, the participants were given different subsequent questions creating subsets of respondents indicated with the letter “s” in the Tables and Figures. All respondents answered the same questions from question 20 onwards (part III).

## Results

Characteristics of the 2158 respondents in the three target groups are given in Table 1. Full details of the questionnaire and summarized data are in the Appendix. As already mentioned, the Bulgarian target group consisted only of parents of young children. The definition of the target groups meant there was an over-representation of females. Due to an unanticipated enthusiastic participation of elderly Belgians, mainly those active on SeniorenNet.be, this subset became substantially larger than the 100 originally planned. Unsurprisingly, there was a high rate of possession of electronic devices, particularly of smartphones. We noticed the relatively strong presence of people with higher education among the respondents in Bulgaria and France.

**Table 1:** Demographic characteristics of questionnaire respondents

	Population characteristics	Belgium		Italy		France		Bulgaria	
		Number	(%)	Number	(%)	Number	(%)	Number	(%)
	**Total**	1204	(100)	308	(100)	300	(100)	346	(100)
**Gender**	Men	496	(41.3)	77	(25.0)	54	(18.0)	54	(15.6)
	Women	708	(58.8)	231	(75.0)	246	(82.0)	292	(84.4)
**Age (years)**	Less than 20	0	(0)	0	(0)	0	(0)	1	(0.3)
	20–29	68	(5.6)	16	(5.2)	24	(8.0)	42	(12.1)
	30–39	106	(8.8)	122	(39.6)	109	(36.3)	183	(52.9)
	40–49	21	(1.7)	55	(17.9)	56	(18.7)	109	(31.5)
	50–59	14	(1.2)	7	(2.3)	7	(2.3)	9	(2.6)
	60–69	423	(35.1)	48	(15.6)	75	(25.0)	2	(0.6)
	70–79	466	(38.7)	47	(15.3)	25	(8.3)	0	(0)
	80 and older	106	(8.8)	13	(4.2)	4	(1.3)	0	(0)
**Education level**	None	2	(0.2)	14	(4.5)	0	(0)	0	(0)
	Primary school	31	(2.6)	30	(9.7)	6	(2.0)	2	(0.6)
	High school	373	(31.0)	114	(37.0)	53	(17.7)	40	(11.6)
	BA^a^ or higher	726	(60.3)	132	(42.9)	212	(70.7)	300	(86.7)
	Other	72	(6.0)	18	(5.8)	29	(9.7)	4	(1.2)
**Owned electronic device (multiple answers possible)**	Smartphone	987	(82.0)	279	(90.6)	284	(94.7)	340	(98.3)
	Tablet	701	(58.2)	171	(55.6)	157	(52.3)	169	(48.8)
	Computer	1118	(92.9)	256	(83.1)	255	(85.0)	316	(91.3)
	None	2	(0.2)	12	(3.9)	4	(1.3)	0	(0)
**Target groups**	Elderly ≥60 years	974	(80.9)	95	(30.8)	100	(33.3)	0	(0)
	Pregnant women	101	(8.4)	108	(35.1)	100	(33.3)	0	(0)
	Parents	129	(10.7)	105	(34.1)	100	(33.3)	346	(100)

^a^Bachelor’s degree or higher

### Responses

As shown in Table 2, responses to Q9 “Did you yourself bring along the vaccine to the person who administered the vaccine?” diverged greatly between countries. Transport of the vaccine by the vaccinee to the healthcare provider (HCP), in a box with its mandatory leaflet, ranged from 4% in the elderly in Italy to 94% in French parents. Within the same country these numbers diverged also between target groups, in Belgium 75% of the elderly brought their vaccine to the HCP compared with 22% of pregnant women, reflecting the organizational diversity of healthcare systems involved in vaccinations.

**Table 2:** Responses to questions 9 (“Did you yourself bring along the vaccine to the person who administered the vaccine?”) and 12 (“Would you have liked to have access to the PPL of that vaccine?")

	Belgium		Italy		France		Bulgaria	
***Q9: Did you yourself bring along the vaccine to the person who administered the vaccine?***								
	**Elderly**							
N=	974		95		100		-	
	Yes	No	Yes	No	Yes	No		
%	75	25	4	96	41	59	na	na
	**Pregnant women**							
N=	101		108		100		-	
	Yes	No	Yes	No	Yes	No		
%	22	78	5	95	36	64	na	na
	**Parents**							
N=	129		105		100		346	
	Yes	No	Yes	No	Yes	No	Yes	No
%	54	47	10	91	94	6	6	94
***Q12: Would you have liked to have access to the PPL of that vaccine?***								
	**Elderly**							
N=	226		92		54		-	
	Yes	No	Yes	No	Yes	No		
%	46	54	32	68	22	78	na	na
	**Pregnant women**							
N=	70		83		55		-	
	Yes	No	Yes	No	Yes	No		
%	30	70	43	57	15	86	na	na
	**Parents**							
N=	57		86		6		246	
	Yes	No	Yes	No	Yes	No	Yes	No
%	37	63	57	43	50	50	80	20

When those who did *not* take the vaccine to the HCP were asked whether they spontaneously received the PPL from the HCP the percentage was generally small: less than 10% for the elderly and parents except for parents in Bulgaria in whom 18% spontaneously received the leaflet. Pregnant women received the leaflet slightly more often: 10% in Belgium, 13% in France and 18% in Italy. A subsequent question revealed that only a small minority, less than 3%, explicitly requested the PPL from the HCP, except for Bulgarian parents of whom 7.5% asked.

These very low numbers contrast markedly with the reported desire for access by those who did not have that access, shown in Table 2 for the different target groups and countries. Lowest percentages were recorded in France, but in the other three countries that percentage was always higher than 30%. When asked why they did not ask for the PPL, the most frequently chosen answer at over 55% was ‘trust in the HCP’ except for pregnant women in Italy in whom it was 40%. The second most checked answer was consistently that they did not know about the possibility of requesting the leaflet (overall average 39%).

In contrast to these expressed desires, proportions with access to the PPL who reported reading it effectively, were low. Belgium and France had highest access to the PPL, 70% and 62%, respectively, but lowest proportions who reported reading the PPL at 25% and 20%. In Italy and Bulgaria 56% and 63% reported reading the PPL, but the actual proportions who had access were only 18% and 29%, respectively, so the overall rate was low in all cases. Among all vaccinees, reading the PPL was a minority occurrence, 18% in Bulgaria and lower elsewhere, assuming that those without access had not read the PPL, contrasting strongly with reported reading of PPL of other medications. On average two-thirds of the respondents reported that they “always” or “regularly” read the PPL and more than 50% did so in all individual target groups in all countries.

We subsequently asked everyone in all target groups about their general use of different sources of information concerning vaccines. Universally, the first source of information was the physician, ranging from 72% in France to 87% in Bulgaria and between 67% and 87% across all the target groups. The internet was frequently the second source, averaging 48% but ranging between 31% and 70% in the target groups. Unsurprisingly, Google was most frequently used for this kind of query with a minimum percentage of 79% in any target group, and was more popular than PPL or pharmacists although the latter results were more divergent among countries and target groups. Pharmacists, with an average of 46% spanned a range from 10% to 67%, in this case among Italian and Belgian parents. Leaflets scored 38%, again with a wide range, from 6% among pregnant women in France to 66% among the same target group in Belgium.

To investigate vaccinees’ understanding of the real nature of the PPL we asked if they knew whether this document was written and approved by the company, or if it was a company-written document approved by a governmental institution. On average two-thirds of the vaccinees gave the correct answer.

Next, following a brief description of the smartphone app currently in use in Spain [49], we asked whether it should be possible to consult vaccine package leaflets electronically. The response in all target groups was overwhelmingly positive: at least 60% of the elderly in all countries agreed and more than 85% in all other target groups, with an average of 79%.

### Electronic format

When asked if they would be willing to download and install an app on their smartphone to access an electronic version of the PPL 64% answered yes, with the scores among the elderly ranging from 34% in France to 59% in Italy. The appeal of information about vaccines in video format was rather modest, averaging 26%. In contrast to the elderly in France and Belgium with 17–18% the elderly in Italy totaled an average of 46%.

**Fig. 2:**
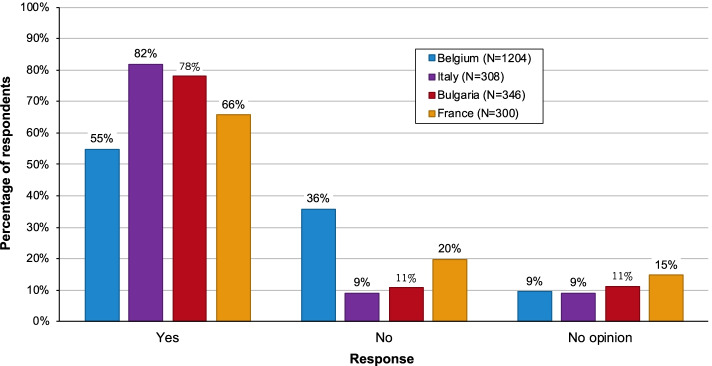
Overall responses by country to the question: *“In your opinion, could the paper package leaflet of a vaccine be replaced by an electronic version (through an app)?”*

**Fig. 3:**
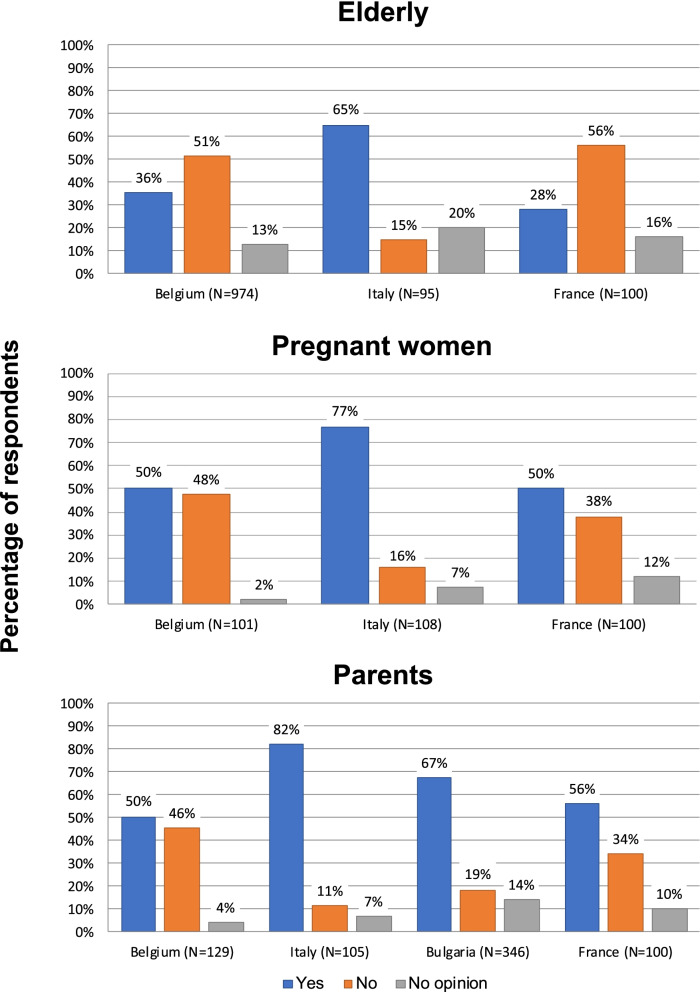
Overall responses by target group and country to the question : *“In your opinion, could the paper package leaflet of a vaccine be replaced by an electronic version (through an app)?”*

**Fig. 4:**
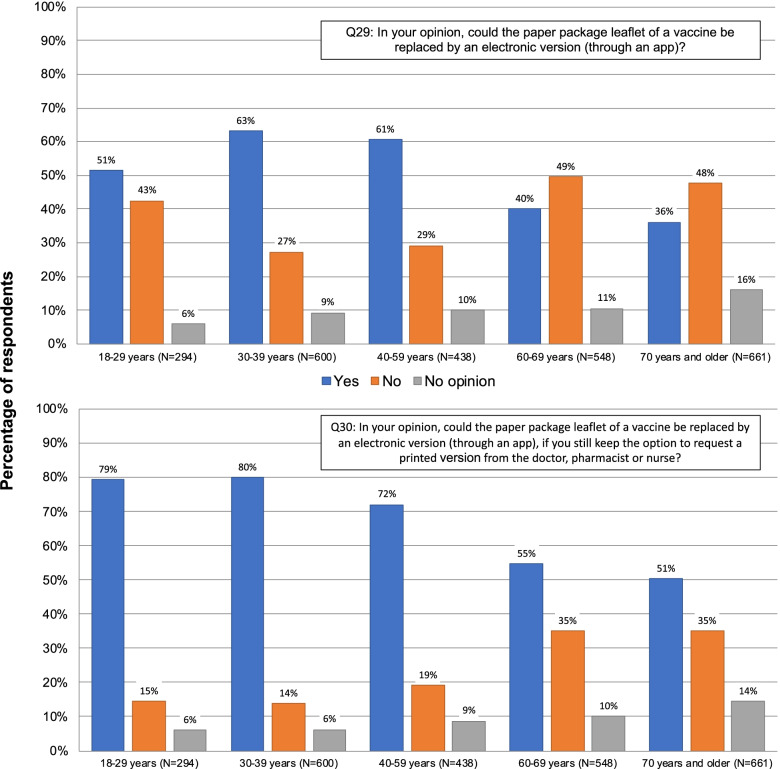
Overall responses by age-group to the questions: *“In your opinion, could the paper package leaflet of a vaccine be replaced by an electronic version (through an app)?”* (upper panel) or *“In your opinion, could the paper package leaflet of a vaccine be replaced by an electronic version (through an app), if you still keep the option to request a printed version from the doctor, pharmacist or nurse?”* (lower panel)

We subsequently asked whether the paper PPL could be replaced by an electronic version. As shown in Fig. 2, there were clear majorities of 75% and 67% in Italy and Bulgaria who responded positively, while in Belgium and France the respondents were more evenly split - 38% and 45% responding yes, and 50% and 43% responding no, respectively. Responses in the different target groups in the different countries are informative and shown in Fig. 3. Pregnant women and parents were more receptive of this replacement and reluctance was mainly centered among the elderly, particularly in France and Belgium. To further characterize the impact of age on this question we analyzed all respondents, including those not included in the target groups, as described in the Methods section, in order to have all age groups represented. In addition, we subdivided the 60+ age population into two groups. The results as presented in Fig. 4 (upper panel) show a clear decrease in willingness from 60 years of age. Perhaps surprising is that almost half of the youngest participants (18–29 years) were unwilling or unsure about accepting an electronic format, but there was a steady increase in acceptance up to 60 years.

We followed-up with precisely the same question with the added possibility of asking a printout of the PPL from the HCP (Fig. 4, lower panel). This had a marked, universal effect of increasing overall willingness to 64%. This was mainly due to increases of 16% in Belgium and 21% in France to achieve a majority willing to accept the switch under these conditions in all countries.

## Discussion

We performed this survey to assess the utility and interest of vaccine recipients or their parents in the information mandated to accompany the vaccine by regulatory practice. As for many recent research projects, the COVID-19 pandemic [45, 50] has had an important impact on the performance of this research. However, rather than postponing the survey we choose to adapt our data-gathering approach to an online environment as the questionnaire itself was readily adaptable, although we are aware that using internet-based technology could bias our results [45, 51, 52]. To limit bias, we used closed internet groups in a time limited way to avoid as far as possible the distribution, redistribution or promotion of our questionnaire-link on open-ended social media such as Facebook.

The COVID-19 pandemic has illustrated the valid and important reasons for our research. The widely disseminated public discussion of availability of COVID-19 vaccines and the need for rapid deployment of vaccines supplied by different manufacturers across the EU with 24 official languages and other legal requirements applicable in each separate EU member state, highlights the consequences of needing a PPL in each box [15, 16]. Even a single vaccine may have a large number of distinctly different packaging versions [53]. This impedes the rapid redistribution of a vaccine among EU member states, an evident necessity in case of an increased regional demand or the existence of localized shortages [54], and is a time-delaying complication for the manufacturer. A similar situation presents itself in other parts of the world [55]. Adding a single, multilingual PPL with each single vaccine dose would result in a larger physical package, entailing higher manufacture, packaging, transportation, refrigeration and storage costs [53].

In these circumstances it is an appropriate time to question the status quo about PPLs in vaccines and other medicines. Change will require both a new legislative and/or regulatory framework for the PPL and a consideration of the actual value for patients or parents these leaflets are intended for. It was in consideration of this latter aspect that we implemented this survey to investigate the actual access, use and opinions about PPLs in a diverse set of vaccinees across a range of European countries. As the balance between utility and cost, both in terms of expense and time for preparation, of the PPL is even more important in the context of increased need for timely vaccination [56], we continued with our survey despite the potential biases inherent to our adapted particular survey method.

Our original intention to conduct face-to-face interviews would have introduced its own kind of bias as many vulnerable people would not have been willing to participate in such interviews during the pandemic. In contrast, although we planned to provide training and guidelines to those who would eventually have interviewed the vaccinees it is likely that using an online questionnaire may have reduced the risk of bias linked to the style and additional instructions provided by the in-person interviewer or the sensitivity of the subject [52]. However, despite the questionnaire explicitly inquiring about personal opinions it is also likely that in an online questionnaire people still answered with elderly acquaintances in mind whom they know to have limited digital skills, as other research suggests [57]. Another potential source of bias in our online survey was the language barrier as this was fixed in the four countries. For this reason, we have not discussed the survey question about the appropriateness or understandability of the PPL language. It is obvious however that an electronic version of the package leaflet could easily, and inexpensively, accommodate multiple European languages as many of these translations already exist. These potential sources of bias increase the importance of selecting different target groups and analyzing the variances between them. In general, despite differences between countries and target groups we discovered consistent trends in the answers that validate our findings.

A key observation from the survey was that patients rarely receive or infrequently read vaccine PPLs across all the settings in marked contrast to leaflets for other medicines that are read much more often [14]. As the number of respondents who read the PPL was rather limited, we do not discuss the expressed opinions about readability and understandability of the leaflet. There is a considerable amount of literature on this topic [14, 25, 58–60] and it is safe to state that the current state of PPLs is sub-optimal.

There is a very high degree of trust in HCPs, particularly general practitioners and pediatricians, who are the most important source of information about vaccines, as reported in other studies [61–63]. We found that HCP rarely use or promote the PPL as a tool for informing the vaccinee, despite this being its primary purpose [15]. We did not investigate the rationale for this but extending existing studies [60] in the context of vaccines does seem to be warranted. In addition, people rarely ask the HCP for the PPL and do not appear to know that they can. Rather than interpreting this as reflective of an attitude of indifference or refusal to be informed, it should be noted that a substantial number of vaccinees clearly expressed the desire to have such access. We can rationalize this attitude by conjecturing that people want easy access, *just in case*, or dislike *not having the possibility* to read the PPL, and the need to promote the existence of the PPL and a vaccinee’s right to read it are important messages in the context of the creation of the informational environment mentioned [9] in the introduction.

An overwhelming majority of respondents believed that the leaflets should be available in an electronic format and many were willing to install an app for this purpose, although the elderly were clearly more reticent to do so. The use of Google (we did not cite other internet search engines) as a generic way of looking up information is fully entrenched and any policy that wants to use the internet to provide information to patients cannot afford to ignore that. Indeed, seeking information about vaccines on the internet was the second most frequently used source, ahead of pharmacists and the PPL.

Finally, we asked about a full switch of the PPL to an electronic version. While most agreed, unsurprisingly this was not the case among the elderly [44]. However, the option of a paper printout, possibly printed at the pharmacy, markedly increased the acceptance of switching to electronic formats to a clear majority in all regions and all target groups. The consistency of these opinions in the different target groups and different regions reinforces the validity of this conclusion and counterbalances some of the biases introduced by the methods used. Nevertheless, we acknowledge that certain vulnerable groups have not participated in our survey and we must be cautious in extrapolating these results to the general public.

Balancing the risks and benefits of switching to electronic leaflets, we believe that with appropriate measures to protect the most vulnerable in society, most notably the option of a paper version, using new technology could contribute to a better informational environment and provide the leaflets with the functionality needed to fulfill their purpose as part of the measures necessary to combat vaccine hesitancy and so improve coverage.

## Conclusions

We believe that the current European regulatory regime mandating the presence of a PPL in the local language with all vaccines does not achieve its purpose of adequately informing the vaccinee. Switching to an electronic format has the potential to enhance the amount and quality of information reaching vaccinees with a small risk of excluding those with low health literacy, poor digital skills, or limited internet access. While the latter would be expected to apply to the elderly, with the widespread use of smartphones we did not find that any age group had particular concerns in having online access; older patients who expressed concerns about a more modern approach were reassured if a printed version would be available on request. Another conclusion is that HCPs do not use the PPL as part of their recognized role to inform their patients. Any change in the regulatory regime of PPLs will therefore need to be accompanied with the necessary guidance and motivation to guarantee that such a change has the full cooperation of HCPs in order to benefit vaccinees.

## Supplementary Information

**Additional file 1.**

## Data Availability

The datasets used and/or analyzed during the current study are available from the corresponding author on reasonable request.

## Abbreviations

PPL: Paper package leaflet(s)
ePL: Electronic package leaflet(s)
HCP: Healthcare provider(s)

## References

[1] Piot P, Larson HJ, O’Brien KL, N’kengasong J, Ng E, Sow S (2019). Immunization: vital progress, unfinished agenda. Nature.

[2] MacDonald NE (2015). Vaccine hesitancy: definition, scope and determinants. Vaccine.

[3] WHO. Ten threats to global health in 2019. https://www.who.int/news-room/spotlight/ten-threats-to-global-health-in-2019. Accessed 4/5/2021.

[4] Donzelli G, Palomba G, Federigi I, Aquino F, Cioni L, Verani M (2018). Misinformation on vaccination: a quantitative analysis of YouTube videos. Hum Vaccin Immunother.

[5] Wang Y, McKee M, Torbica A, Stuckler D (2019). Systematic literature review on the spread of health-related misinformation on social media. Soc Sci Med.

[6] McKee M, Middleton J (2019). Information wars: tackling the threat from disinformation on vaccines. BMJ.

[7] Smith N, Graham T (2019). Mapping the anti-vaccination movement on Facebook. Inf Commun Soc.

[8] Kata A (2010). A postmodern Pandora’s box: anti-vaccination misinformation on the internet. Vaccine.

[9] Gostin LO, Hodge JG, Bloom BR, El-Mohandes A, Fielding J, Hotez P (2020). The public health crisis of underimmunisation: a global plan of action. Lancet Infect Dis.

[10] Ames HM, Glenton C, Lewin S. Parents’ and informal caregivers’ views and experiences of communication about routine childhood vaccination: a synthesis of qualitative evidence. Cochrane Database Syst Rev. 2017;2.2:CD011787. 10.1002/14651858.CD011787.pub2 PMC5461870 28169420

[11] Brewer NT, Chapman GB, Rothman AJ, Leask J, Kempe A (2017). Increasing vaccination: putting psychological science into action. Psychol Sci Public Interest.

[12] Raynor DT (2018). Written information on medicines for patients: learning from the PIL. Drug Ther Bull.

[13] Committee EP (1998). A guideline on the readability of the label and package leaflet of medicinal products for human use.

[14] Nathan JP, Zerilli T, Cicero LA, Rosenberg JM (2007). Patients’ use and perception of medication information leaflets. Ann Pharmacother.

[15] Europea XU (2004). Directive 2004/27/EC of the European Parliament and of the Council of 31 March 2004 amending Directive 2001/83/EC on the Community code relating to medicinal products for human use. Official J Eur Union L.

[16] Anonymous DIRECTIVE, 2001/83/EC. OF THE EUROPEAN PARLIAMENT AND OF THE COUNCIL of 6 November 2001 on the Community code relating to medicinal products for human use, 2020. https://eur-lex.europa.eu/legal-content/ENG/TXT/PDF/?uri=CELEX:02001L0083-20121116&from=EN. Accessed 4/5/2021.

[17] Anonymous, Product information: Reference documents and guidelines, 2018. https://www.ema.europa.eu/en/human-regulatory/marketing-authorisation/product-information/product-information-reference-documents-guidelines. Accessed 4/5/2021.

[18] Freeman AL (2019). How to communicate evidence to patients. Drug Ther Bull.

[19] Jose J (2020). Communication on drug safety-related matters to patients: is it even more significant in this digital era?. Ther Adv Drug Saf.

[20] Young A, Tordoff J, Smith A (2018). Regulatory agencies’ recommendations for medicine information leaflets: are they in line with research findings?. Res Social Adm Pharm.

[21] Raynor DT, Svarstad B, Knapp P, Aslani P, Rogers MB, Koo M (2007). Consumer medication information in the United States, Europe, and Australia: a comparative evaluation. J Am Pharm Assoc.

[22] Maat HP, Lentz L (2010). Improving the usability of patient information leaflets. Patient Educ Couns.

[23] Pires C, Vigário M, Cavaco A (2015). Readability of medicinal package leaflets: a systematic review. Rev Saude Publica.

[24] Grime J, Blenkinsopp A, Raynor DK, Pollock K, Knapp P (2007). The role and value of written information for patients about individual medicines: a systematic review. Health Expect.

[25] Carrigan N, Raynor DK, Knapp P (2008). Adequacy of patient information on adverse effects: an assessment of patient information leaflets in the UK. Drug Saf.

[26] St-Amour M, Guay M, Perron L, Clément P, Baron G, Petit G (2006). Are vaccination information leaflets useful for vaccinators and parents?. Vaccine.

[27] Van Dijk L, Monteiro SP, Vervloet M, de Bie J, Raynor D. Study on the package leaflets and the summaries of product characteristics of medicinal products for human use. PIL’s Study European Union; 2014. https://ec.europa.eu/health/sites/health/files/files/committee/75meeting/pil_s.pdf. Accessed 4/5/2021.

[28] Thomas TM, Pollard AJ (2020). Vaccine communication in a digital society. Nat Mater.

[29] Betsch C, Brewer NT, Brocard P, Davies P, Gaissmaier W, Haase N (2012). Opportunities and challenges of Web 2.0 for vaccination decisions. Vaccine.

[30] Madrigal-Cadavid J, Amariles P, Pino-Marín D, Granados J, Giraldo N (2020). Design and development of a mobile app of drug information for people with visual impairment. Res Soc Adm Pharm.

[31] Ciciriello S, Johnston RV, Osborne RH, Wicks I, deKroo T, Clerehan R (2013). Multimedia educational interventions for consumers about prescribed and over-the‐counter medications. Cochrane Database Syst Rev.

[32] Roberts K, Thakkar R, Autor D, Bisordi F, Fitton H, Garner C (2020). Creating E-labeling platforms: an industry vision. Clin Pharmacol Ther.

[33] Koumoundourou M, Koutsabasis P, Darzentas JS. Informing the design of mobile device-based patient instructions leaflets: the case of Fentanyl patches. Future Focused Thinking - DRS International Conference. Brighton; 2016. https://dl.designresearchsociety.org/drs-conference-papers/drs2016/researchpapers/140/. Accessed 4/5/2021.

[34] Hansen C, Interrante JD, Ailes EC, Frey MT, Broussard CS, Godoshian VJ (2016). Assessment of YouTube videos as a source of information on medication use in pregnancy. Pharmacoepidemiol Drug Saf.

[35] Berkman ND, Sheridan SL, Donahue KE, Halpern DJ, Viera A, Crotty K (2011). Health literacy interventions and outcomes: an updated systematic review. Evid Rep Technol Assess (Full Rep).

[36] Agencia Española de Medicamentos y Productos Sanitarios. - La AEMPS informa - Notas informativas - 2016 - El Centro de Información online de Medicamentos Autorizados de la AEMPS (CIMA) galardonado en la categoría de Premio Ciudadanía 2015. https://www.aemps.gob.es/en/informa/notasInformativas/laAEMPS/2016/NI-AEMPS_06-2016-CIMA-premio-cuidadania.htm. Accessed 4/5/2021.

[37] Electronic product information for human medicines in the EU: key principles A joint EMA-HMA-EC collaboration. 2020. https://www.ema.europa.eu/en/documents/regulatory-procedural-guideline/electronic-product-information-human-medicines-european-union-key-principles_en.pdf. Accessed 4/5/2021.

[38] Wilsdon T, Lawlor R, Li L, Rafila A, García Rojas A (2020). The impact of vaccine procurement methods on public health in selected European countries. Exp Rev Vacc.

[39] Dyda A, King C, Dey A, Leask J, Dunn AG (2020). A systematic review of studies that measure parental vaccine attitudes and beliefs in childhood vaccination. BMC Public Health.

[40] Sadaf A, Richards JL, Glanz J, Salmon DA, Omer SB (2013). A systematic review of interventions for reducing parental vaccine refusal and vaccine hesitancy. Vaccine.

[41] Frew PM, Chung Y, Fisher AK, Schamel J, Basket MM (2016). Parental experiences with vaccine information statements: Implications for timing, delivery, and parent-provider immunization communication. Vaccine.

[42] Arcury TA, Sandberg JC, Melius KP, Quandt SA, Leng X, Latulipe C (2020). Older adult internet use and eHealth literacy. J Appl Gerontol.

[43] Onyeaka HK, Romero P, Healy BC, Celano CM. Age differences in the use of health information technology among adults in the United States: an analysis of the Health Information National Trends Survey. J Aging Health. 2021;33:147–54. 10.1177/0898264320966266 33031007

[44] Gordon NP, Crouch E (2019). Digital information technology use and patient preferences for internet-based health education modalities: cross-sectional survey study of middle-aged and older adults with chronic health Conditions. JMIR Aging.

[45] Kalaycioglu O (2020). Guidance for research on the COVID-19 disease in times of pandemic. J Health Soc Sci.

[46] Qualtrics. https://www.qualtrics.com/. Accessed 4/5/2021.

[47] IBM Corp. Released 2019. IBM SPSS Statistics for Windows, Version 26.0. Armonk, NY: IBM Corp.

[48] Ermenlieva NM, Tsankova GS, Todorova TT (2019). Seasonal influenza vaccination: knowledge, attitude and practice in Varna, Bulgaria. Ther Adv Vaccines Immunother.

[49] Anonymous. Medicine online information center of AEMPS - CIMA https://cima.aemps.es/cima/publico/home.html. Accessed 4/5/2021.

[50] Ledford H (2020). Coronavirus shuts down trials of drugs for multiple other diseases. Nature.

[51] Fricker RD, Schonlau M (2002). Advantages and disadvantages of Internet research surveys: evidence from the literature. Field Methods.

[52] Evans JR, Mathur A. The value of online surveys. Internet research. 2005.

[53] Anonymous. From vaccines shortages to sustainable vaccine supply. Vaccines Europe Position., 2019. https://www.vaccineseurope.eu/wp-content/uploads/2019/04/VE-Paper_shortagesFIN.pdf. Accessed 4/5/2021.

[54] Filia A, Rota M, Grossi A, Martinelli D, De Graaf T, Dominguez A (2020). Are vaccine shortages a relevant public health issue in Europe?. Eur J Public Health.

[55] Hinman AR, Orenstein WA, Santoli JM, Rodewald LE, Cochi SL (2006). Vaccine shortages: history, impact, and prospects for the future. Annu Rev Public Health.

[56] Grady C, Shah S, Miller F, Danis M, Nicolini M, Ochoa J, et al. So much at stake: ethical trade-offs in accelerating SARS-CoV-2 vaccine development. Vaccine. 2020. 10.1016/j.vaccine.2020.08.017 PMC7418641 32826103

[57] Hammar T, Nilsson A-L, Hovstadius B (2016). Patients’ views on electronic patient information leaflets. Pharm Pract (Granada).

[58] Schwappach DL, Mülders V, Simic D, Wilm S, Thürmann PA (2011). Is less more? Patients’ preferences for drug information leaflets. Pharmacoepidemiol Drug Saf.

[59] Burgers C, Beukeboom CJ, Sparks L, Diepeveen V (2015). How (not) to inform patients about drug use: use and effects of negations in Dutch patient information leaflets. Pharmacoepidemiol Drug Saf.

[60] Young A, Tordoff J, Leitch S, Smith A (2019). Patient-focused medicines information: general practitioners’ and pharmacists’ views on websites and leaflets. Health Educ J.

[61] Sabaté LR, Diego L. Are we offering patients the right medicines information? A retrospective evaluation of readability and quality in online patient drug information. Eur J Hosp Pharm. 2020.

[62] Hesse BW, Nelson DE, Kreps GL, Croyle RT, Arora NK, Rimer BK (2005). Trust and sources of health information: the impact of the internet and its implications for health care providers: findings from the first Health Information National Trends Survey. Arch Int Med.

[63] Mononen N. From paper to cyber: medicines Information as a strategic goal in Finland and the European Union. 2020.

