# Supplementary material for: Replacing vaccine paper package inserts: a multi-country questionnaire study on the acceptability of an electronic replacement in different target groups
Source: BMC Public Health. 2022 Jan 24;22:156. doi: 10.1186/s12889-022-12510-8 (PMC8785016; doi:10.1186/s12889-022-12510-8)
Supplement: Supplementary file 1 — Additional file 1. [file 12889_2022_12510_MOESM1_ESM.pdf]

Replacing vaccine paper package inserts: a multi-country questionnaire study on the acceptability of an electronic replacement in different target groups

Martina Bamberger*, Hans De Loof*, Charlotte Marstboom, Stéphanie Oury, Paolo Bonanni, Odile Launay, Mira Kojouharova, Pierre Van Damme

Corresponding author: Martina.Bamberger@uantwerpen.be

Supplementary Materials

Responses to questions

Q10: If you answered no to Q9, did you yourself bring along the vaccine to the person who administered the vaccine? Page 2

Q11: Have you requested and received the paper package leaflet of that vaccine from the person who administered it? Page 2

Q12: If you answered no to Q11, would you have liked to have access to the paper leaflet of that vaccine? Page 3

Q14: Have you read the paper leaflet of that vaccine? Page 3

Q15: If you answered yes to Q14, are the leaflets written in the language you would like to read them in? Page 4

Q16: If you answered yes to Q14, was the leaflet in a format that was easy to read? Page 4

Q17: If you answered yes to Q14, was the leaflet easy to understand? Page 5

Q18: If you answered yes to Q14, did the leaflet provide you with the information you needed? Page 5

Q21: Do you look on the internet for the text of the package leaflet of vaccines? Page 6

Q22: Do you know where on the internet you can find the text of the package leaflet of medicines and vaccines? Page 6

Q26: Should it be possible for you to consult the package leaflet of a vaccine electronically? Page 7

Q27: Are you prepared to download a free app to electronically read the package leaflet of a vaccine? Page 7

Q28: Would you like to receive the information of the package leaflet of vaccines in a video format? Page 8

Q29: In your opinion, could the paper package leaflet of a vaccine be replaced by an electronic version (through an app)? Page 8

Full questionnaire Pages 9-14

	BELGIUM		ITALY		FRANCE		BULGARIA	
Q10: If you answered no to Q9, did you yourself bring along the vaccine to the person who administered the vaccine?								
**Elderly**								
N=	245 (s)		91 (s)		60 (s)		-	
	Yes	No	Yes	No	Yes	No		
n (%)	17 (6.9)	228 (93.1)	5 (5.5)	86 (94.5)	5 (8.3)	55 (91.7)	na	na
**Pregnant women**								
N=	79 (s)		103 (s)		64 (s)		-	
	Yes	No	Yes	No	Yes	No		
n (%)	8 (10.1)	71 (89.9)	18 (17.5)	85 (82.5)	8 (12.5)	56 (87.5)	na	na
**Parents**								
N=	60 (s)		95 (s)		6 (s)		326 (s)	
	Yes	No	Yes	No	Yes	No	Yes	No
n (%)	3 (5.0)	57 (95.0)	8 (8.4)	87 (91.6)	0 (0)	6 (100)	60 (18.4)	266 (81.6)
Q11: Have you requested and received the paper package leaflet of that vaccine from the person who administered it?								
**Elderly**								
N=	228 (s)		86 (s)		55 (s)		-	
	Yes	No	Yes	No	Yes	No		
n (%)	2 (0.9)	226 (99.1)	1 (1.2)	85 (98.8)	1 (1.8)	54 (98.2)	na	na
**Pregnant women**								
N=	71 (s)		85 (s)		56 (s)		-	
	Yes	No	Yes	No	Yes	No		
n (%)	1 (1.4)	70 (98.6)	2 (2.4)	83 (97.6)	1 (1.8)	55 (98.2)	na	na
**Parents**								
N=	57 (s)		87 (s)		6 (s)		266 (s)	
	Yes	No	Yes	No	Yes	No	Yes	No
n (%)	0 (0)	57 (100)	1 (1.1)	86 (98.9)	0 (0)	6 (100)	20 (7.5)	246 (92.5)

(s) indicates sub-set

	BELGIUM		ITALY		FRANCE		BULGARIA	
Q12: If you answered no to Q11, would you have liked to have access to the paper leaflet of that vaccine?								
**Elderly**								
N=	226 (s)		85 (s)		54 (s)		-	
	Yes	No	Yes	No	Yes	No		
n (%)	104 (46.0)	122 (54.0)	27 (31.8)	58 (68.2)	12 (22.2)	42 (77.8)	na	na
**Pregnant women**								
N=	70 (s)		83 (s)		55 (s)		-	
	Yes	No	Yes	No	Yes	No		
n (%)	21 (30.0)	49 (70.0)	36 (43.4)	47 (56.6)	8 (14.5)	47 (85.5)	na	na
**Parents**								
N=	57 (s)		86 (s)		6 (s)		246 (s)	
	Yes	No	Yes	No	Yes	No	Yes	No
n (%)	21 (36.8)	36 (63.2)	49 (57.0)	37 (43.0)	3 (50.0)	3 (50.0)	196 (79.7)	50 (20.3)

	BELGIUM			ITALY			FRANCE			BULGARIA		
Q14: Have you read the paper leaflet of that vaccine?												
**Elderly**												
N=	748 (s)			10 (s)			46 (s)			-		
	Yes	No	Uncertain*	Yes	No	Uncertain*	Yes	No	Uncertain*			
n (%)	186 (24.9)	512 (68.4)	50 (6.7)	5 (50.0)	5 (50.0)	0 (0)	18 (39.1)	27 (58.7)	1 (2.2)	na	na	na
**Pregnant women**												
N=	31 (s)			25 (s)			45 (s)			-		
	Yes	No	Uncertain*	Yes	No	Uncertain*	Yes	No	Uncertain*			
n (%)	8 (25.8)	23 (74.2)	0 (0)	21 (84.0)	4 (16.0)	0 (0)	4 (8.9)	39 (86.7)	2 (4.4)	na	na	na
**Parents**												
N=	72 (s)			19 (s)			94 (s)			100 (s)		
	Yes	No	Uncertain*	Yes	No	Uncertain*	Yes	No	Uncertain*	Yes	No	Uncertain*
n (%)	15 (20.8)	50 (69.4)	7 (9.7)	4 (21.1)	12 (57.1)	3 (15.8)	15 (16.0)	72 (76.6)	7 (7.4)	63 (63.0)	16 (16.0)	21 (21.0)

(s) indicates subset * Could not remember

	BELGIUM		ITALY		FRANCE		BULGARIA	
Q15: If you answered yes to Q14, are the leaflets written in the language you would like to read them in?								
**Elderly**								
N=	186 (s)		5 (s)		18 (s)		-	
	Yes	No	Yes	No	Yes	No		
n (%)	126 (67.7)	60 (32.3)	5 (100)	0 (0)	14 (77.8)	4 (22.2)	na	na
**Pregnant women**								
N=	8 (s)		21 (s)		4 (s)		-	
	Yes	No	Yes	No	Yes	No		
n (%)	8 (100)	0 (0)	21 (100)	0 (0)	3 (75.0)	1 (25.0)	na	na
**Parents**								
N=	15 (s)		4 (s)		15 (s)		63 (s)	
	Yes	No	Yes	No	Yes	No	Yes	No
n (%)	12 (80.0)	3 (20.0)	4 (100)	0 (0)	12 (80.0)	3 (20.0)	52 (82.5)	11 (17.5)
Q16: If you answered yes to Q14, was the leaflet in a format that was easy to read?								
**Elderly**								
N=	186 (s)		5 (s)		18 (s)		-	
	Yes	No	Yes	No	Yes	No		
n (%)	126 (67.7)	60 (32.3)	5 (100)	0 (0)	14 (77.8)	4 (22.2)	na	na
**Pregnant women**								
N=	8 (s)		21 (s)		4 (s)		-	
	Yes	No	Yes	No	Yes	No		
n (%)	8 (100)	0 (0)	21 (100)	0 (0)	3 (75.0)	1 (25.0)	na	na
**Parents**								
N=	15 (s)		4 (s)		15 (s)		63 (s)	
	Yes	No	Yes	No	Yes	No	Yes	No
n (%)	12 (80.0)	3 (20.0)	4 (100)	0 (0)	12 (80.0)	3 (20.0)	52 (82.5)	11 (17.5)

(s) indicates sub-set

	BELGIUM		ITALY		FRANCE		BULGARIA	
Q17: If you answered yes to Q14, was the leaflet easy to understand?								
**Elderly**								
N=	186 (s)		5 (s)		18 (s)		-	
	Yes	No	Yes	No	Yes	No		
n (%)	147 (79.0)	39 (21.0)	4 (80.0)	4 (20.0)	14 (77.8)	4 (22.2)	na	na
**Pregnant women**								
N=	8 (s)		21 (s)		4 (s)		-	
	Yes	No	Yes	No	Yes	No		
n (%)	7 (87.5)	1 (12.5)	21 (100)	0 (0)	2 (50.0)	2 (50.0)	na	na
**Parents**								
N=	15 (s)		4 (s)		15 (s)		63 (s)	
	Yes	No	Yes	No	Yes	No	Yes	No
n (%)	12 (80.0)	3 (20.0)	2 (50.0)	2 (50.0)	11 (73.3)	4 (26.7)	53 (84.1)	10 (15.5)
Q18: If you answered yes to Q14, did the leaflet provide you with the information you needed?								
**Elderly**								
N=	186 (s)		5 (s)		18 (s)		-	
	Yes	No	Yes	No	Yes	No		
n (%)	165 (88.7)	21 (11.3)	4 (80.0)	4 (20.0)	17 (94.4)	1 (5.6)	na	na
**Pregnant women**								
N=	8 (s)		21 (s)		4 (s)		-	
	Yes	No	Yes	No	Yes	No		
n (%)	8 (100)	0 (0)	21 (100)	0 (0)	4 (100)	0 (0)	na	na
**Parents**								
N=	15 (s)		4 (s)		15 (s)		63 (s)	
	Yes	No	Yes	No	Yes	No	Yes	No
n (%)	12 (80.0)	3 (20.0)	3 (75.0)	1 (25.0)	13 (87.6)	2 (13.3)	51 (81.0)	12 (19.0)

(s) indicates sub-set

	BELGIUM		ITALY		FRANCE		BULGARIA	
Q21: Do you look on the internet for the text of the package leaflet of vaccines?								
**Elderly**								
N=	974		95		100		-	
	Yes	No	Yes	No	Yes	No		
n (%)	334 (34.3)	640 (65.7)	23 (24.2)	72 (75.8)	24 (24.0)	76 (76.0)	na	na
**Pregnant women**								
N=	101		21		4		-	
	Yes	No	Yes	No	Yes	No		
n (%)	7 (87.5)	1 (12.5)	21 (100)	0 (0)	2 (50.0)	2 (50.0)	na	na
**Parents**								
N=	129		105		100		346	
	Yes	No	Yes	No	Yes	No	Yes	No
n (%)	64 (49.6)	65 (50.4)	47 (44.8)	58 (55.20)	39 (39.0)	61 (61.0)	215 (62.1)	131 (37.9)
Q22: Do you know where on the internet you can find the text of the package leaflet of medicines and vaccines?								
**Elderly**								
N=	974		95		100		-	
	Yes	No	Yes	No	Yes	No		
n (%)	659 (67.7)	315 (32.3)	43 (45.3)	52 (54.7)	35 (35.0)	65 (65.0)	na	na
**Pregnant women**								
N=	101		108		100		-	
	Yes	No	Yes	No	Yes	No		
n (%)	79 (78.2)	22 (21.8)	94 (87.0)	14 (13.0)	60 (60.0)	40 (40.0)	na	na
**Parents**								
N=	129		105		100		346	
	Yes	No	Yes	No	Yes	No	Yes	No
n (%)	93 (72.1)	36 (27.9)	67 (63.8)	38 (36.2)	58 (58.0)	42 (42.0)	249 (72.0)	97 (28.0)

	BELGIUM			ITALY			FRANCE			BULGARIA		
Q26: Should it be possible for you to consult the package leaflet of a vaccine electronically?												
**Elderly**												
N=	974			95			100			-		
	Yes	No	No opinion	Yes	No	No opinion	Yes	No	No opinion			
n (%)	686 (70.4)	129 (13.2)	1590 (16.3)	65 (68.4)	7 (7.4)	23 (24.2)	61 (61.0)	32 (32.0)	7 (7.0)	na	na	na
**Pregnant women**												
N=	101			108			100			-		
	Yes	No	No opinion	Yes	No	No opinion	Yes	No	No opinion			
n (%)	86 (85.1)	3 (3.0)	12 (11.9)	96 (88.9)	6 (5.6)	6 (5.6)	87 (87.0)	8 (8.0)	5 (5.0)	na	na	na
**Parents**												
N=	129			105			100			346		
	Yes	No	Uncertain*	Yes	No	Uncertain*	Yes	No	Uncertain*	Yes	No	Uncertain*
n (%)	116 (89.9)	3 (2.3)	10 (7.8)	93 (88.6)	3 (2.9)	9 (8.6)	87 (87.0)	6 (6.0)	7 (7.0)	327 (94.5)	4 (1.2)	15 (4.3)
Q27: Are you prepared to download a free app to electronically read the package leaflet of a vaccine?												
**Elderly**												
N=	974			95			100					
	Yes	No	No opinion	Yes	No	No opinion	Yes	No	No opinion			
n (%)	559 (57.4)	270 (27.7)	145 (14.9)	56 (58.9)	27 (28.4)	12 (12.6%)	34 (34.0)	57 (57.0)	9 (9.0)	na	na	na
**Pregnant women**												
N=	101			108			100					
	Yes	No	No opinion	Yes	No	No opinion	Yes	No	No opinion			
n (%)	75 (74.3)	19 (18.8)	7 (6.9)	108 (100)	0 (0)	0 (0)	52 (52.0)	37 (37.0)	11 (11.0)	na	na	na
**Parents**												
N=	129			105			100			346		
	Yes	No	No opinion	Yes	No	No opinion	Yes	No	No opinion	Yes	No	No opinion
n (%)	87 (67.4)	28 (21.7)	14 (10.9)	92 (87.6)	8 (7.6)	5 (4.8)	66 (66.0)	21 (21.0)	13 (13.0)	245 (70.8)	52 (15.0)	49 (14.2)

	BELGIUM			ITALY			FRANCE			BULGARIA		
Q28: Would you like to receive the information of the package leaflet of vaccines in a video format?												
**Elderly**												
N=	974			95			100			-		
	Yes	No	No opinion	Yes	No	No opinion	Yes	No	No opinion			
n (%)	169 (17.4)	559 (57.4)	245 (25.3)	44 (46.3)	34 (35.80)	17 (17.9)	18 (18.0)	70 (70.0)	12 (12.0)	na	na	na
**Pregnant women**												
N=	101			108			100			-		
	Yes	No	No opinion	Yes	No	No opinion	Yes	No	No opinion			
n (%)	12 (11.9)	80 (79.2)	9 (8.9)	61 (56.5)	33 (30.6)	14 (13.0)	19 (19.0)	65 (65.0)	16 (16.0)	na	na	na
**Parents**												
N=	129			105			100			346		
	Yes	No	Uncertain*	Yes	No	Uncertain*	Yes	No	Uncertain*	Yes	No	Uncertain*
n (%)	11 (8.5)	103 (79.8)	15 (11.6)	60 (57.1)	27 (25.7)	18 (17.1)	42 (42.0)	44 (44.0)	14 (14.0)	116 (33.5)	160 (46.2)	70 (20.2)
Q29: In your opinion, could the paper package leaflet of a vaccine be replaced by an electronic version (through an app)?												
**Elderly**												
N=	974			95			100					
	Yes	No	No opinion	Yes	No	No opinion	Yes	No	No opinion			
n (%)	346 (35.5)	501 (51.4)	127 (13.0)	62 (65.2)	14 (14.7)	19 (20.0)	28 (28.0)	56 (56.0)	16 (16.0)	na	na	na
**Pregnant women**												
N=	101			108			100					
	Yes	No	No opinion	Yes	No	No opinion	Yes	No	No opinion			
n (%)	51 (50.5)	48 (47.5)	2 (2.0)	83 (76.9)	17 (15.7)	8 (7.4)	50 (50.0)	38 (38.0)	12 (12.0)	na	na	na
**Parents**												
N=	129			105			100			346		
	Yes	No	No opinion	Yes	No	No opinion	Yes	No	No opinion	Yes	No	No opinion
n (%)	65 (50.4)	59 (45.7)	5 (3.9)	86 (81.9)	12 (11.4)	7 (6.7)	56 (56.0)	34 (34.0)	10 (10.0)	233 (67.3)	64 (18.5)	49 (14.2)

FULL QUESTIONNAIRE

If **you or your child** received a vaccination in the **past two years**, we want to ask you a few questions about the paper package leaflet inside the box of vaccines and medicines.

PART I: Personal questions

1) Gender:

1.1 Male 0

1.2 Female 0

2) 2.1 Nationality: …………………….

3) 3.1 Age: ………..

4) What is your highest level of education?

4.1 None 0

4.2 Primary school 0

4.3 High school 0

4.4 Bachelor's degree or higher 0

4.5 Other: … 0

5) Do **you** have a job or had training in health care?

5.1 Yes 0

5.2 No 0

6) Do **you** have (have access to) a smartphone, a tablet or a computer? (Multiple answers are possible)

6.1 Smartphone 0

6.2 Tablet 0

6.3 Computer 0

6.4 None 0

7) If you are a woman: Are **you** pregnant **and** have **you** been vaccinated during your current pregnancy? **(If you are a man go directly to question 8)**

7.1 Yes 0 → **Go to PART II,** to the leaflet questions **about the vaccination during your pregnancy**

7.2 No 0

<u>**8)** If you answered **No** (to question 7)</u>

Do **you** have children who have been vaccinated **in your presence** in the last 2 years?

8.1 Yes 0 → **Go to PART II,** to the leaflet questions **about this(!) vaccination**

8.2 No 0 → **Go to PART II,** to the leaflet questions and answer about **your** latest vaccination

PART II: Package leaflet (vaccine leaflet specific, most recent vaccination)

Remember these questions are about the paper package **leaflet** of the **vaccine** used **in that last vaccination.**

9) Did **you** yourself bring along the vaccine to the person who administered the vaccine? (to you or your child)

9.1 Yes 0 → **Go to question nr 14**

9.2 No 0

<u>If you answered **no**:</u>

10) Did **you** spontaneously receive the package leaflet of that vaccine from the person who administered it?

10.1 Yes 0 → **Go to question nr 14**

10.2 No 0

11) Have **you** requested **and** received the paper package leaflet of that vaccine from the person who administered it?

11.1 Yes 0 → **Go to question nr 14**

11.2 No 0

<u>If you answered **no:**</u>

12) Would **you** have liked to have access to the paper leaflet of that vaccine?

12.1 Yes 0

12.2 No 0

13) Why did **you** not request it? (Multiple answers are possible)

13.1 Trust in the person who injected the vaccine 0

13.2 No interest / no need 0

13.3 I have already read it in the past 0

13.4 I do not understand leaflets 0

13.5 I did not know I could ask for it

13.6 Other reason: ……. 0

→ **Go to PART III**

14) Have **you** read the paper leaflet of that vaccine?

14.1 Yes 0

14.2 No 0

14.3 I don't remember 0 → **Go to PART III**

<u>If you answered **Yes (to question 14)**:</u>

15) Are the leaflets written in the language you would like to read them in?

15.1 Yes 0

15.2 No 0

16) Was the leaflet in a format that was easy **to read**?

16.1 Yes 0

16.2 No 0

17) Was the leaflet easy **to understand**?

17.1 Yes 0

17.2 No 0

18) Did the leaflet provide **you** with the information you needed?

18.1 Yes 0

18.2 No 0

If you answered **No (to question 14)** (Multiple answers are possible):

19) The reason:

19.1 Trust in the person who injected the vaccine 0

19.2 No interest / no need 0

19.3 I have already read it in the past 0

19.4 I do not understand leaflets 0

19.5 I use another source of information 0

19.6 Other reason: ……. 0

PART III: General questions/preferences about leaflets

We will now ask you a number of questions about package leaflets **in general.**

20) Do **you** read the **paper** package leaflet for medicines?

20.1 Always 0

20.2 Regularly 0

20.3 Sometimes 0

20.4 Never 0

21) Do **you** look on the internet for the text of the package leaflet of vaccines?

21.1 Yes 0

21.2 No 0

22) Do **you** know **where** on the internet you can find the text of the package leaflet of medicines and vaccines?

22.1 Yes 0

22.2 No 0

23) If **you** want to know more about vaccines in general, what source of information do you use? (Multiple answers are possible)

23.1 Doctor 0

23.2 Pharmacist 0

23.3 Nurse 0

23.4 Other medical personnel 0

23.5 Family member 0

23.6 Internet 0

23.7 Package leaflet 0

23.8 Others… 0

24) If **you** look for information about vaccines on the internet, which statement applies for **you**? (Multiple answers are possible; possible to not reply)

24.1 I look this up on Google 0

24.2 I look this up on social media sites, e.g. Facebook 0

24.3 I search with a specific app on my smartphone 0

Which app? …………

24.4 I look this up on a specific site 0

Which site? …………

25) Which of these two statements is **true**?

25.1 The paper package leaflet of medicines and vaccines is made by the company and approved by a governmental institution. 0

25.2 The paper package leaflet of medicines and vaccines is made by the company and approved by the company. 0

25.3 I don't know. 0

PART IV: Electronic leaflet

In Spain there is an app with the information from the paper package leaflets. Everybody can freely download this app. It has a search bar, but you can also scan the barcode of the medicine or vaccine. The app will then instantly provide the current information about this particular medicine or vaccine. This information is also available on tablets and on a personal computer.

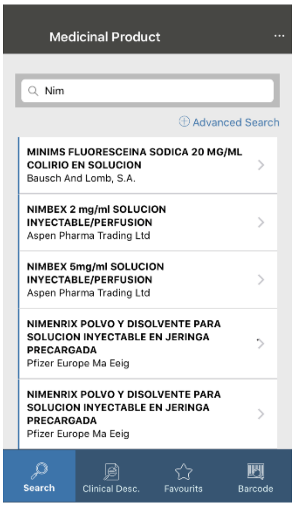

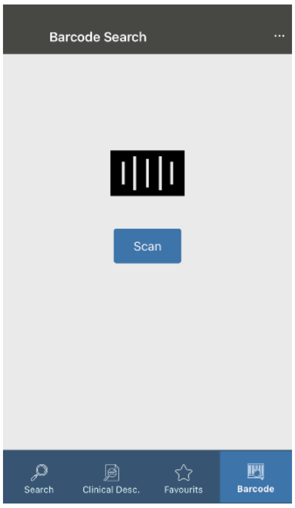

The following questions are about the package leaflet **information of vaccines in general**.

26) Should it be possible for **you** to consult the package leaflet of a vaccine electronically?

26.1 Yes 0

26.2 No 0

26.3. No opinion 0

27) Are **you** prepared to download a free app to electronically read the package leaflet of a vaccine?

27.1 Yes 0

27.2 No 0

27.3 No opinion 0

28) Would **you** like to receive the information of the package leaflet of vaccines in a video format?

28.1 Yes 0

28.2 No 0

28.3 No opinion 0

29) In your opinion, could the paper package leaflet of a vaccine be replaced by an electronic version (through an app)?)?

29.1 Yes 0

29.2 No 0

29.3 No opinion 0

30) In your opinion, could the paper package leaflet of a vaccine be replaced by an electronic version (through an app), if you still keep the option to request a printed version from the doctor, pharmacist or nurse?

30.1 Yes 0

30.2 No 0

30.3 No opinion 0
